# Genetic Variability, Genotype × Environment Interaction, Correlation, and GGE Biplot Analysis for Grain Iron and Zinc Concentration and Other Agronomic Traits in RIL Population of Sorghum (*Sorghum bicolor* L. Moench)

**DOI:** 10.3389/fpls.2017.00712

**Published:** 2017-05-05

**Authors:** Rahul M. Phuke, Kotla Anuradha, Kommineni Radhika, Farzana Jabeen, Ghanta Anuradha, Thatikunta Ramesh, K. Hariprasanna, Shivaji P. Mehtre, Santosh P. Deshpande, Gaddameedi Anil, Roma R. Das, Abhishek Rathore, Tom Hash, Belum V. S. Reddy, Are Ashok Kumar

**Affiliations:** ^1^International Crops Research Institute for the Semi-Arid TropicsHyderabad, India; ^2^Professor Jayashankar Telangana State Agricultural UniversityHyderbad, India; ^3^Indian Institute of Millets ResearchHyderabad, India; ^4^Marathwada Agricultural UniversityParbhani, India

**Keywords:** sorghum, micronutrients, iron and zinc, recombinant inbred lines, genotype × environment interaction, GGE biplot

## Abstract

The low grain iron and zinc densities are well documented problems in food crops, affecting crop nutritional quality especially in cereals. Sorghum is a major source of energy and micronutrients for majority of population in Africa and central India. Understanding genetic variation, genotype × environment interaction and association between these traits is critical for development of improved cultivars with high iron and zinc. A total of 336 sorghum RILs (Recombinant Inbred Lines) were evaluated for grain iron and zinc concentration along with other agronomic traits for 2 years at three locations. The results showed that large variability exists in RIL population for both micronutrients (Iron = 10.8 to 76.4 mg kg^−1^ and Zinc = 10.2 to 58.7 mg kg^−1^, across environments) and agronomic traits. Genotype × environment interaction for both micronutrients (iron and zinc) was highly significant. GGE biplots comparison for grain iron and zinc showed greater variation across environments. The results also showed that G × E was substantial for grain iron and zinc, hence wider testing needed for taking care of G × E interaction to breed micronutrient rich sorghum lines. Iron and zinc concentration showed high significant positive correlation (across environment = 0.79; *p* < 0.01) indicating possibility of simultaneous effective selection for both the traits. The RIL population showed good variability and high heritabilities (>0.60, in individual environments) for Fe and Zn and other traits studied indicating its suitability to map QTL for iron and zinc.

## Introduction

Dietary deficiency of micronutrients, leading to hidden hunger, has been recognized by the World Health Organization as a serious human health problem worldwide (World Health Organization, 2002), especially in populations having limited access to fruits, vegetables and livestock products. Three micronutrients iron (Fe), zinc (Zn) and provitamin A are widely deficient, especially among low economic group population in developing countries. Fe and Zn deficiencies are most prevalent with almost over three billion people affected word wide (Welch and Graham, 2004). Nearly 500,000 children (<5 years of age) die annually because of Zn and Fe deficiencies (Black et al., 2008). Among the 26 major risk factors of the global burden of disease estimates, iron deficiency ranks 9th, while zinc, and vitamin A deficiencies ranks at 11th and 13th positions, respectively (Ezzati et al., 2002). Deficiency of iron and zinc results in poor growth, reduced immunity, fatigue, irritability, weakness, hair loss, wasting of muscles, sterility, morbidity and even death in acute cases (Haas and Brownlie, 2001; Pfeiffer and McClafferty, 2007; Stein, 2010). Traditional efforts to address the problem of micronutrient deficiency have focused on micronutrient supplementation and food fortification (White and Broadley, 2005; Ghandilyan et al., 2006). However, these methods have not proven to be sustainable, especially in developing countries where people cannot afford fortified products with a high micronutrient content and these countries do not have logistics to supply the supplemented foods. Instead, most people in these regions consume cereals as their staple food, which provide only a small amount of the micronutrients and do not meet human nutrition needs (Shi et al., 2008). Also associated costs and small number of primary health care programs in developing countries makes micronutrient supplementation and food fortification as difficult task. An alternative (or complement) to the above approaches is to use plant breeding to naturally fortify commonly consumed staple crops with micronutrients, through a process known as genetic biofortification (Bouis, 2003). Agriculture is the primary source of nutrients necessary for a healthy life, but most agricultural policies and technologies have focused on improving profitability at the farm and agroindustry levels, not on improving nutrition (Bouis and Welch, 2010). Given the prevalence of hidden hunger, there is growing interest for agriculture to play a role in improving nutrition, in particular by paying more attention to the nutritional quality of food. Biofortification is a scientific method for improving nutritional value of foods already consumed by those suffering from hidden hunger (Bouis et al., 2011).

The disease burden related to iron deficiency in India could be reduced by 19–58% by crop biofortification (Stein et al., 2008). More recently Meenakshi et al. (2010) concluded that overall biofortification can make a significant impact on reducing the burden of micronutrient deficiencies in the developing world in a highly cost-effective manner. Biofortification is being used to improve micronutrient intake of populations in many parts of the world and was recently ranked as the fifth most cost-effective solution for the world's greatest problems by Copenhagen Consensus Centre (2008). Hence there is an urgent and compelling need to develop varieties with improved concentration of micronutrients using biofortification.

Sorghum is among top 10 crops that feed the world (Ashok Kumar et al., 2015). Its nutritional richness and stress tolerance makes it an important crop choice in Africa and Asia. Sorghum is the second cheapest source of energy and micronutrients (after pearl millet); and a vast majority of the population in Africa and central India depend on sorghum for their dietary energy and micronutrient requirement (Rao et al., 2006). Sorghum stover is the major source of dry fodder for urban and peri-urban dairy production in India (Tesfaye, 1998). In terms of nutrient uptake, sorghum account for about 35% of total intake of calories, protein, Fe and Zn in dominant production/consumption regions of India (Rao et al., 2006) and in low income group populations, it goes beyond 50% of the micronutrient requirement. Biofortification of sorghum by increasing mineral micronutrients (especially iron and zinc) in grains is widespread interest (Pfeiffer and McClafferty, 2007). Breeding for grain Fe and Zn enrichment requires sufficient genetic variability for grain micronutrient in available germplasm as well as information on genetic control of grain micronutrient content in the seed. Significant positive association reported earlier (Reddy et al., 2010) between two traits indicates common genomic region or genes or biochemical pathway involved in expression of the trait. Hence the knowledge of phenotypic association between traits gives basic idea for simultaneous improvement of the traits. Apart from this, understanding environment is considered important in breeding for traits that depends on many factors (Campbell and Lafever, 1980; Ghaderi et al., 1980; Fox and Rosielle, 1982; Yau et al., 1991; Joshi et al., 2007). Proper characterization and understanding of locations is very important for screening breeding lines of greater concentration of zinc and iron in grain (Ortiz-Monasterio et al., 2007). Soil micronutrient status vary greatly in dry lands where sorghum cultivation is concentrated, under such conditions, genotype × environment (G × E) interaction for agronomic and grain nutrient traits expect to be large and may not permit differentiation of performance of genotypes across environments.

Many tools and technique have been suggested for characterizing and grouping environments, with biplot analysis considered the most valuable. Interpretation of performance of number of genotypes in a broad range of environments is generally affected by large G × E interactions (Gauch and Zobel, 1996). Analysis of variance describes only main effects; it tests the significance of the G × E interaction but do not provide insight into particular pattern of genotype or environment that give rise to G × E interaction. A type of linear bilinear model suitable for grouping sites and cultivars without cultivar rank change is the site regression model (SREG). The model is also named as GGE (Yan et al., 2001) because it includes the effect of genotype plus G × E interaction. Biplots obtained from graphing first two components of the multiplicative part of SREG (genotype plus G × E interaction) are useful for summarizing data (Gabriel, 1971, 1978). Very few attempts have been made to identify G × E interaction for grain Fe and Zn in sorghum. In this study we have examined 2 years data from three locations for grain micronutrients (Fe and Zn) and other agronomic traits to evaluate genetic variability, G × E interaction and association of grain Fe and Zn and other agronomic traits using RILs (Recombinant Inbred Lines) of sorghum.

## Materials and methods

### Experiment material and multi-environment field trials (MET)

Present investigation was carried out principally to study the variation for grain iron (Fe) and zinc (Zn) concentration, G × E interaction and association between these two traits and agronomic traits in RIL population consisting of 334 individuals of F_6_ generation developed using two contrasting parents 296B and PVK801 (Table 1).

**Table 1:** **Characteristics of the parents used in mapping population (RIL) development**.

**Sr. No**.	**Parent**	**Pedigree**	**Days to 50% flowering**	**Fe (mgKg^−1^)**	**Zn (mgKg^−1^)**	**Grain Mold reaction**	**Plant Height (cm)**
1	296B	IS 3922 (Kafir-durra) × Karad Local (Kharif local)	85	32	20	Susceptible	110
2	PVK801	[(IS 23528 × SPV 475) x (PS 29154)]-4-2-2-4 4	80	42	30	Resistant	140

Phenotypic trials were conducted for 2 years (November 2012 to March 2014) at three locations viz., International Crops Research Institute for the Semi-Arid Tropics (ICRISAT) located at an altitude of 545 m above mean sea level with latitude of 17.53° N and longitude of 78.27° E, Indian Institute of Millets Research (IIMR) located at an altitude of 542 m above mean sea level with latitude of 17.19° N and longitude of 78.28° E and Vasantrao Naik Marathwada Krishi Vidhyapeeth (VNMKV) Parbhani located at an altitude of 357 m above mean sea level with latitude of 18.45° N and longitude of 76.13° E in India. All the experiments were conducted in post-rainy seasons to obtain best quality grain for assessing grain Fe and Zn. For first year (Post-rainy 2012–13) based on availability of seed only 309 RILs along with Parents (296B and PVK 801) were used for sowing at three locations, and in second year (Post-rainy 2013–14) based on availability of seed, 334 RILs at ICRISAT and 325 RILs at IIMR and VNMKV were planted along with parents (Table 2). All trials were conducted using Alpha Lattice design with three replications. Seeds of each entry were distributed equally to seed packets, representing number of rows of each plot size; and then randomized plot numbers were assigned to each plot seed packets and arranged according to planned field layout.

**Table 2:** **Field layouts and Sowing for each location in two consecutive year with three replications**.

**Year**	**Location**	**Plots/rep**	**RIL + Parents**	**Design**	**Planting date**	**Soil type**
Post-rainy 2012–13	ICRISAT 2012–13 (E1)	320	309 RIL + P1 (6 reps) + P2 (5 reps) = 320	(10 entries/block × 32 blocks) × 3 reps	Late November	Shallow black soil
	IIMR 2012–13 (E2)	320	309 RIL + P1 (6 reps) + P2 (5 reps) = 320	(10 entries/block × 32 blocks) × 3 reps	Late November	Shallow black soil
	VNMKV 2012–13 (E3)	324	309 RIL + P1 (8 reps) + P2 (7 reps) = 324	(9 entries/block × 36 blocks) × 3 reps	Late November	Deep black soil
Post-rainy 2013–14	ICRISAT 2013–14 (E4)	360	334 RIL + P1 (13 reps) + P2 (13 reps) = 360	(10 entries/block × 36 blocks) × 3 reps	Middle September	Shallow black soil
	IIMR 2013–14 (E5)	360	325 RIL + P1 (17 reps) + P2 (18 reps) = 360	(10 entries/block × 36 blocks) × 3 reps	Late September	Shallow black soil
	VNMKV 2013–14 (E6)	360	325 RIL + P1 (17 reps) + P2 (18 reps) = 360	(18 entries/block × 20 blocks) × 3 reps	Late September	Deep black soil

### Agronomic practices and data recording

Trial was sown by tractor-mounted 2-cone planter (7100 US model) at ICRISAT while hand sowing was done at IIMR and VNMKV, with each entry planted in two rows of 2 m length, Overplanted plots were thinned 15 days after planting to single plants, spaced 10 cm apart within each row. Soil Fe and Zn contents were analyzed by DTPA extractable method at Charles Renard Analytical Laboratory, ICRISAT, Patancheru, and expressed as mg kg-1 (ppm). These Fe and Zn contents in the soil were in the sufficient range for normal plant requirements (2.6 to 4.5 mg kg^−1^ for Fe; 0.6 to 1.0 mg kg^−1^ for Zn). The crop was supplied with a fertilizer dose of 80 kg N and 40 kg P2O_5_ per hectare and nitrogen was applied in two split doses half as basal and remaining half at 35 days after sowing. Trials were irrigated as needed, to ensure no moisture stress. All recommended agronomic practices were followed for raising a good crop. Observations were recorded for days to 50% flowering, plant height, 100-grain weight (Test weight), grain yield and grain Fe and Zn concentration. The entries were harvested at physiological maturity. During harvest, main panicles of five random plants from each plot were harvested and stored separately in a cloth bag to produce clean grain samples for micronutrient analysis. Remaining panicles of plot were harvested as a bulk. These panicles were sundried for 10–15 days. While threshing, five separately harvested panicles were manually threshed first and approximately 20 g of grains were collected for Fe and Zn analysis, and left over grains from these panicles were added to the bulk grain produced by threshing in a multi head machine thresher. Grain yield including the 20 g sample taken for micronutrient analysis was recorded for each plot and extrapolate to tonne per hectare for grain yield analysis. In all experiments, self-pollinated (SP) grain samples were used to estimate grain Fe and Zn concentration expressed in mg kg^−1^. At the time of harvesting, 5 representative main panicles from each plot were harvested at physiological maturity (120–150 days after planting) for assessing grain micronutrients. Harvested panicles were put directly in a separate cloth bag to avoid soil contamination and dried them in the sun to <12% post-harvest grain moisture content. Grains were cleaned from glumes, panicle chaff and debris and transferred to new non-metal fold envelops and stored in cold temperature. Care was taken at each step to avoid contamination of grains with dust particles and any other extraneous matter (Stangoulis and Sison, 2008).

Grain Fe and Zn concentrations from the all three locations were analyzed at the Charles Renard Analytical Laboratory, ICRISAT-Patancheru, India following the method described by Wheal et al. (2011). The ground grain samples were digested in closed tubes; and Fe and Zn in the digests were analyzed using Inductively Coupled Plasma Optical Emission Spectrometry (ICP-OES).

### Statistical analysis

#### Analysis of variance

Combined Analysis of Variance was carried out at three locations across 2 years by modeling individual environment (a combination of location and year) error variances using mixed model procedure. Five variance components (σ^2^g, σ^2^gy, σ^2^gl, σ^2^gyl, σ^2^e) were estimated for each of the six traits studied using restricted maximum likelihood (Paterson and Thompson, 1971) estimation procedure of GenStat Software, 17th edition (VSN International, Hemel Hempstead, UK). In these analyses, location and year was fitted as a fixed effect. Genotype, blocks, replicates and genotype interactions with location and year, were fitted as random effects. Heterogeneous error variances of individual year and location in combined analysis is incorporated by error variance modeling using mixed model analysis The phenotypic observations *z*_*ijklm*_ on accession *m* in replicate *k* of block *l* of location *j* and year *i* was modeled as:

$$\begin{array}{l} z_{ijklm}=\mu +y_{i}+e_{j}+ye_{ij}+r_{ijk}+b_{ijkl}+g_{m}+{\left(yg\right)}_{im}+{\left(eg\right)}_{jm}\\ +{\left(yeg\right)}_{ijm}+\varepsilon_{ijklm} \end{array}$$

Where μ is the grand mean; *y*_*i*_ is the fixed effect of year *i*; *e*_*j*_ is the fixed effect of location *j*; *ye*_*ij*_ is the fixed effect of interaction between year *i* and location *j*; *g*_*m*_ is the random effect of genotype m and is ~NID(0, $\sigma_{g}^{2}$); *r*_*ijk*_ is the random effect of replication in location *j* and year *i* and is ~ NID(0, $\sigma_{r}^{2}$); *b*_*ijkl*_ is the random effect of block *l* nested with replication *k* in location *j* and year *i* and is ~ NID(0, $\sigma_{b}^{2}$); (*yg*)_*im*_ is the random effect of the interaction between genotype *m* and year *i* and is ~NID(0, $\sigma_{yg}^{2}$); (*eg*)_*jm*_ is the random effect of the interaction between accession *m* in location *j* and ~ NID(0, $\sigma_{eg}^{2}$); (*yeg*)_*ijm*_ is the random effect of the interaction effect of the genotype *m* in year *i* and location j and ~ NID(0, $\sigma_{yg}^{2}$); and ε_*ijklm*_ is the random residual effect and ~ NID(0, $\sigma_{\varepsilon }^{2}$).

Analysis of variance were also conducted using data from each environment for all six traits.

Heritability (H^2^, broad sense) at individual environment was estimated from analysis of variance. The formula used was-

$$\begin{array}{l} H^{2}=\frac{\sigma_{g}^{2}}{\sigma_{g}^{2}\;+\;\sigma_{\varepsilon }^{2}/r} \end{array}$$

Whereas Heritability (H^2^) estimates across environments were estimated by the formula-

$$\begin{array}{l} H^{2}=\frac{\sigma_{g}^{2}}{\sigma_{g}^{2}\;+\;\sigma_{yg}^{2}/y\;+\;\sigma_{eg}^{2}/l\;+\;\sigma_{yeg}^{2}/yl\;+\;\sigma_{\varepsilon }^{2}/ryl} \end{array}$$

where *r, y, l* denotes the number of replicates, years and environments respectively.

#### GGE biplots model

The basic model for GGE biplot is based on site regression analysis and is given by:

$$\begin{array}{l} Y_{ij}-\mu -\beta_{j}=\overset{K}{\underset{k=1}{\sum }}\lambda_{k}\xi_{ik}\eta_{jk}\;+\;\varepsilon_{ij} \end{array}$$

Where *Y*_*ij*_ = the mean yield of genotype i (= 1,2,…,g) in environment j (= 1,2,…e), μ = the grand mean, β_*j*_ = the main effect of environment j, (μ + β_*j*_) = mean yield of environment *j*, λ_*k*_ = the singular value (SV) of *k*^*th*^ principal component (PC), ξ_*ik*_ = the eigen-vector of genotype i for PC_*k*_, η_*jk*_ = the eigen-vector of environment j for PC_*k*_, K is the number of PC axes retained in the model (K ≤ min (g,e) and *K* = 2 for a 2-dimensional biplot) and ε_*ij*_ = the residual associated with genotype i in environment j.

In present study, heritability adjusted-genotype main effect plus genotype-environment interaction (HA-GGE) biplot (Yan and Holland, 2010) was used to understand the G × E interaction, identify the superior genotypes across environment and evaluate the test environments based on representativeness and discrimination power on genotypic differences.

#### Association between grain micronutrients and agronomic traits

Relationship between grain Fe and Zn concentration and agronomic traits like days to 50% flowering, plant height, 100-seed weight and grain yield were evaluated using Pearson correlation coefficient using BLUPs (Best Linear Unbiased Predictors) of single environment as well as across the environments.

## Results

The RIL population derived from cross 296 B × PVK 801 having 334 RILs of F_6_ generation were phenotyped over three locations for 2 years for agronomic traits and grain Fe and Zn concentration along with parents to obtain means and variances. Phenotypic data collected for population during post-rainy seasons 2012–13 and 2013–14 at three different locations were analyzed statistically to obtain variance components. Hereafter, referred as E_1_ (ICRISAT 2012–13), E_2_ (IIMR 2012–13), E_3_ (VNMKV 2012–13), E_4_ (ICRISAT 2013–14), E_5_ (IIMR 2013–14), and E_6_ (VNMKV 2013–14).

### Mean performance

The means, standard deviation, ranges and significance of genotypes for traits measured for RILs were compared with parental means in all environment separately and summarized in (Table 3).

**Table 3:** **Descriptive statistics of phenotypic values in RILs derived from a cross 296B X PVK 801 in three locations for two seasons, post-rainy 2012–13 and 2013–14**.

**Trait**	**Environment**	**296B (P1)**		**PVK 801 (P2)**		**RILs**		**S.D**	**P1 vs. P2**	**P1 vs. RIL**	**P2 vs. RIL**
		**MEAN**	**RANGE**	**MEAN**	**RANGE**	**MEAN**	**RANGE**		**Pr > F**	**Pr > F**	**Pr > F**
DTF	ICRISAT 2012–13 (E1)	82.00	80–85	78.00	75–80	80.00	70–92	3.12	^**^	^**^	^**^
	IIMR 2012–13 (E2)	78.00	72–89	73.00	66–78	76.00	65–88	4.79	^*^	NS	NS
	VNMKV 2012–13 (E3)	84.00	81–88	82.00	81–84	84.00	77–91	2.23	NS	^*^	NS
	ICRISAT 2013–14 (E4)	90.00	85–97	83.00	77–89	87.00	74–101	3.89	^**^	^*^	^**^
	IIMR 2013–14 (E5)	91.00	85–99	88.00	79–86	90.00	77–100	3.78	^**^	^*^	NS
	VNMKV 2013–14 (E6)	88.00	83–92	84.00	80–87	86.00	78–97	3.26	^**^	^**^	^**^
PH (cm)	ICRISAT 2012–13 (E1)	119.44	100–140	163.33	150–180	146.27	90–230	26.98	^**^	^**^	^**^
	IIMR 2012–13 (E2)	106.05	97–114	156.20	142–169	133.28	75–208	26.31	^**^	^**^	^**^
	VNMKV 2012–13 (E3)	94.56	87–102	152.76	140–170	126.57	66–195	24.82	^**^	^**^	^**^
	ICRISAT 2013–14 (E4)	105.47	95–122	128.73	117–143	121.34	65–198	21.51	^**^	^**^	^**^
	IIMR 2013–14 (E5)	105.93	85–133	142.11	121–162	131.00	57–196	22.95	^**^	^**^	^**^
	VNMKV 2013–14 (E6)	99.40	85–110	145.11	134–156	125.32	68–200	25.06	^**^	^**^	^**^
TW (g)	ICRISAT 12-13 (E1)	3.07	2.7–3.5	3.77	3.0–4.5	3.22	2.0–4.9	0.42	^**^	NS	^**^
	IIMR 2012–13 (E2)	2.60	2.0–3.5	2.58	2.0–3.5	2.53	1.2–4.2	0.47	NS	NS	NS
	VNMKV 2012–13 (E3)	2.24	1.8–2.6	2.77	2.2–3.4	2.27	1.3–3.2	0.34	^**^	NS	^**^
	ICRISAT 2013–14 (E4)	2.90	2.0–4.2	3.26	2.5–4.2	2.77	1.2–4.3	0.50	^*^	NS	^**^
	IIMR 2013–14 (E5)	2.90	2.1–3.9	3.61	2.4–4.7	2.92	1.3–4.9	0.52	^**^	NS	^**^
	VNMKV 2013–14 (E6)	2.76	2.2–3.8	3.13	2.0–3.7	2.73	1.4–3.9	0.42	^**^	NS	^**^
Fe (mg kg^−1^)	ICRISAT 2012–13 (E1)	28.00	24–32	33.40	29.4–41.0	33.60	16.5–65.2	5.60	^**^	^**^	NS
	IIMR 2012–13 (E2)	28.50	22–35	33.00	26.0-36.7	33.00	19.3–56.3	6.34	^*^	^**^	NS
	VNMKV 2012–13 (E3)	46.33	38–49	49.40	44.0–66.0	49.26	33.0–76.4	6.93	^*^	^**^	NS
	ICRISAT 2013–14 (E4)	26.00	20–31	28.20	20.8–41.3	28.00	15.0–47.6	4.90	^**^	^*^	NS
	IIMR 2013–14 (E5)	30.80	21–40	35.90	30.2–52.4	35.85	19.8–50.1	5.08	^*^	^*^	NS
	VNMKV 2013–14 (E6)	27.24	19–37	33.60	22.3–44.3	34.00	10.8–67.3	7.89	^*^	^*^	NS
Zn (mg kg^−1^)	ICRISAT 2012–13 (E1)	21.32	19–26	24.33	21.0–30.9	24.63	13.6–54.2	4.78	^**^	^**^	NS
	IIMR 2012–13 (E2)	21.00	17–24	22.00	20.6–23.6	24.76	13.9–44.3	5.00	^**^	^**^	^*^
	VNMKV 2012–13 (E3)	26.44	20–36	30.43	23.0–37.0	31.43	17.3–58.7	6.46	^*^	^**^	NS
	ICRISAT 2013–14 (E4)	14.63	10–18	16.46	14.0–21.7	17.33	10.2–33.0	3.50	^**^	^**^	NS
	IIMR 2013–14 (E5)	21.19	13–29	24.82	18.3–40.0	25.66	11.8–41.2	4.03	^**^	^*^	^**^
	VNMKV 2013–14 (E6)	19.69	14–24	24.06	19.0–33.6	24.72	11.8–46.5	5.27	^*^	^**^	NS
GY (t ha^−1^)	ICRISAT 2012–13 (E1)	4.13	2.5–5.6	5.85	5.0–7.3	3.70	0.7–7.0	0.11	^**^	NS	^**^
	IIMR 2012–13 (E2)	3.00	1.7–1.4	3.60	2.8–4.5	2.70	0.4–6.5	0.10	NS	NS	^**^
	VNMKV 2012–13 (E3)	1.40	0.8–2.0	2.39	1.8–3.0	1.50	0.3–3.0	0.05	^**^	NS	^**^
	ICRISAT 2013–14 (E4)	2.80	1.4–3.8	4.16	2.2–6.2	3.10	0.4–5.4	0.07	^**^	NS	^**^
	IIMR 2013–14 (E5)	2.14	1.0–3.8	5.19	1.8–3.0	3.30	0.25–6.4	0.12	^**^	NS	^**^
	VNMKV 2013–14 (E6)	2.69	1.4–3.0	3.99	2.2–7.8	2.50	0.4–5.1	0.09	^**^	NS	^**^

*DTF, Days to 50% flowering; PH, Plant Height (cm); TW, 100 seed weight (g); Fe, grain iron concentration (mg kg^−1^); Zn, grain zinc concentration (mg kg^−1^), and GY, Grain Yield (t ha^−1^); SD, Standard Deviation*.

* Significant at 5% level;

** *Significant at 1% level, NS, Non-significant*.

Except for days to 50% flowering (DTF), female parent 296 B exhibited lower means as compared to another parent PVK 801 for all agronomic traits in all six environments. But parental means difference for DTF in E_3_ was non-significant, similarly 100 seed weight and grain yield for E_2_ was non-significant, whereas for remaining all traits two parents were significantly differed in all six environments. Mean performance for grain iron and zinc concentration in both parents were higher in E_3_, whereas mean performance of iron and zinc for both parents was lowest in E_4_, also both parents exhibited wider range of variation for grain iron and zinc studied in different environments. The mean performance of parents for other agronomic traits like plant height, 100 seed weight and grain yield was higher in E_1_.

Mean performance of RILs for grain iron and zinc was highest in E_3_ whereas lowest in E_4_, for other agronomic traits like plant height, 100 seed weight and grain yield the RIL performance was high in E_1_, whereas mean performance of RIL for 100 seed weight and grain yield was the lowest in E_3_. The mean performance of RIL population for grain iron and zinc was significantly different than 296B over all six environments, whereas except E_2_ and E_5_ for zinc, the mean performances of RILs for grain iron and zinc was non-significant. For 100 seed weight and grain yield the RIL population was non-significantly differed from 296B, whereas except for 100 seed weight in E_2_ RIL population was significantly differed from PVK801.

### Genotypic variance and G × E interaction

Grain micronutrient (iron and zinc) concentration showed highly significant genotypic variances in all individual environments (Table 4). Grain iron concentration has showed the highest genotypic variance in E_6_ and grain zinc concentrations showed the highest variance in E_3_. Same trend was continued in across environments (Table 5). Both the traits showed highly significant genotypic variances, whereas genotype × year (σ^2^gy) interaction for zinc was significant, but less in magnitude compared to genotypic variance. For iron, genotype × year (σ^2^gy) interaction was non-significant. Genotype × location (σ^2^gl) interactions were found to be non-significant for both micronutrients but genotype × year × location (σ^2^gyl) interactions were highly significant for both the traits, also the magnitude of variance was more than genotypic variances.

**Table 4:** **Genotypic variance (σ^**2**^g), standard error (SE) and heritability in broad-sense (H^**2**^) for traits in 296 B × PVK 801-derived RIL population at six different environments**.

**Trait**	**ICRISAT 2012–13 (E1)**			**IIMR 2012–13 (E2)**			**VNMKV 2012–13 (E3)**		
	**σ^2^g**	**SE**	**H^2^**	**σ^2^g**	**SE**	**H^2^**	**σ^2^g**	**SE**	**H^2^**
DTF	6.34^**^	0.60	0.85	14.46^**^	1.36	0.90	2.70^**^	0.26	0.83
PH	667.30^**^	55.41	0.97	658.21^**^	53.90	0.98	590.11^**^	47.86	0.99
TW	0.10^**^	0.01	0.83	0.15^**^	0.01	0.88	0.09^**^	0.01	0.95
Fe	15.51^**^	1.68	0.78	20.79^**^	2.17	0.81	27.44^**^	2.85	0.80
Zn	10.00^**^	1.12	0.74	12.72^**^	1.33	0.80	23.41^**^	2.43	0.80
GY (t ha^−1^)	0.02^**^	0.00	0.90	0.014^**^	0.00	0.95	0.003^**^	0.00	0.94
**Trait**	**ICRIS0AT 2013–14 (E4)**			**IIMR 2013–14 (E5)**			**VNMKV 2013–14 (E6)**		
	σ^2^**g**	**SE**	**H**^2^	σ^2^**g**	**SE**	**H**^2^	σ^2^**g**	**SE**	**H**^2^
DTF	10.48^**^	0.91	0.89	7.34^**^	0.75	0.81	7.97^**^	0.68	0.91
PH	407.21^**^	33.20	0.95	486.60^**^	39.51	0.97	618.32^**^	48.87	0.99
TW	0.12^**^	0.01	0.78	0.13^**^	0.01	0.78	0.14^**^	0.01	0.93
Fe	8.72^**^	1.05	0.68	10.84^**^	1.30	0.68	31.36^**^	3.23	0.77
Zn	5.48^**^	0.60	0.73	4.60^**^	0.68	0.56	12.71^**^	1.40	0.74
GY (t ha^−1^)	0.013^**^	0.00	0.89	0.02^**^	0.00	0.93	0.027^**^	0.00	0.80

*DTF, Days to 50% flowering; PH, Plant Height (cm); TW, 100 seed weight (g); Fe, grain iron concentration (mg kg^−1^); Zn, grain zinc concentration (mg kg^−1^), and GY, Grain Yield (t ha^−1^)*.

^*^Significant at 5% level;

** *Significant at 1% level*.

**Table 5:** **Genotypic variance (σ^**2**^g), Genotype × Year (σ^**2**^gy), Genotype × Location (σ^**2**^gl), Genotype × Year × Location (σ^**2**^gyl) interactions, standard error (SE) and operational heritability's (h^**2**^, broad-sense) for traits in 296 B × PVK 801-derived RIL population**.

**Trait**	**Pooled**								
	**σ^2^g**	**SE**	**σ^2^gy**	**SE**	**σ^2^gl**	**SE**	**σ^2^gyl**	**SE**	**h^2^**
DTF	4.33^**^	0.41	−0.115 NS	0.15	−0.016 NS	0.198	3.49^**^	0.279	0.86
PH	500.54^**^	40.08	5.74 NS	2.92	−0.03 NS	3.07	63.15^**^	4.35	0.97
TW	0.06^**^	0.01	0.00 NS	0.00	0.006 NS	0.00	0.06^**^	0.00	0.79
Fe	4.18^**^	0.69	−0.17 NS	0.66	−0.7 NS	0.80	14.32^**^	1.18	0.58
Zn	4.17^**^	0.51	0.71^**^	0.35	−0.14 NS	0.37	5.22^**^	0.54	0.69
GY (t ha^−1^)	0.22^**^	0.02	0.0043 NS	0.01	0.05^**^	0.017	0.3003^*^	0.212	0.73

*DTF, Days to 50% flowering; PH, Plant Height (cm); TW, 100 seed weight (g); Fe, grain iron concentration (mg kg^−1^); Zn, grain zinc concentration (mg kg^−1^), and GY, Grain Yield (t ha^−1^); SD, Standard Deviation*.

* Significant at 5% level;

** *Significant at 1% level; NS, Non-significant*.

The analysis of variance from our experimentation showed highly significant differences among genotypes (RILs) for all six traits in all individual environments (Table 4) as well as across the environments (Table 5). Agronomic trait such as days to 50% flowering, plant height, 100-seed weight and grain yield showed highly significant genotypic variances (σ^2^g) in all environments, same trend was continued for across environment analysis. For days to 50% flowering and 100-seed weight, the highest genetic variance was found in E_2_ and for plant height in E_1_ and grain yields in E_6_. For all agronomic traits, genotype × year (σ^2^gy) interactions were found non-significant and for genotype × location (σ^2^gl) interactions, except for grain yield all agronomic traits were found non-significant. Whereas, for all agronomic traits the genotype × year × location (σ^2^gyl) interactions were highly significant, but in lesser magnitude compared to genotypic variance (σ^2^g).

### Estimation of heritability

In present study all traits were highly heritable (>0.60) as per scale of Robinson (1966) in individual environments except for grain zinc concentration in E_5_ (Table 4). Agronomic traits like days to 50% flowering, plant height, 100-seed weight and grain yield were found to be more heritable than grain micronutrients. However, a partitioned genotype by environment interaction component reduced the heritability for across environments (pooled analysis). Broad sense heritability for all traits was high (0.30–0.60) across six environments (Table 5). Broad sense heritability using pooled data ranged from 0.58 (iron) to 0.96 (plant height), plant height was the most heritable trait in all the environments. For grain iron and zinc concentration, the heritability was high in first year (post-rainy 2012–13) compared to second year (post-rainy 2013–14), whereas environment wise E_5_ showed the lowest value for iron and zinc heritability in tune with low genotypic variance for these traits in same environment.

### Heritability-adjusted GGE biplots

#### Test environment evaluation based on heritability-adjusted GGE biplots

Availability of multiyear and multilocation data facilitates to compare the test environments for studied traits. Using HA-GGE biplot analysis for grain micronutrients, first two principal component explained 59.49% and 77.86% of G + G × E interaction variation for grain iron and zinc concentration respectively, whereas HA-GGE biplot for grain yield depicted 76.91% of the G + G × E interaction variation.

**Figure 1:**
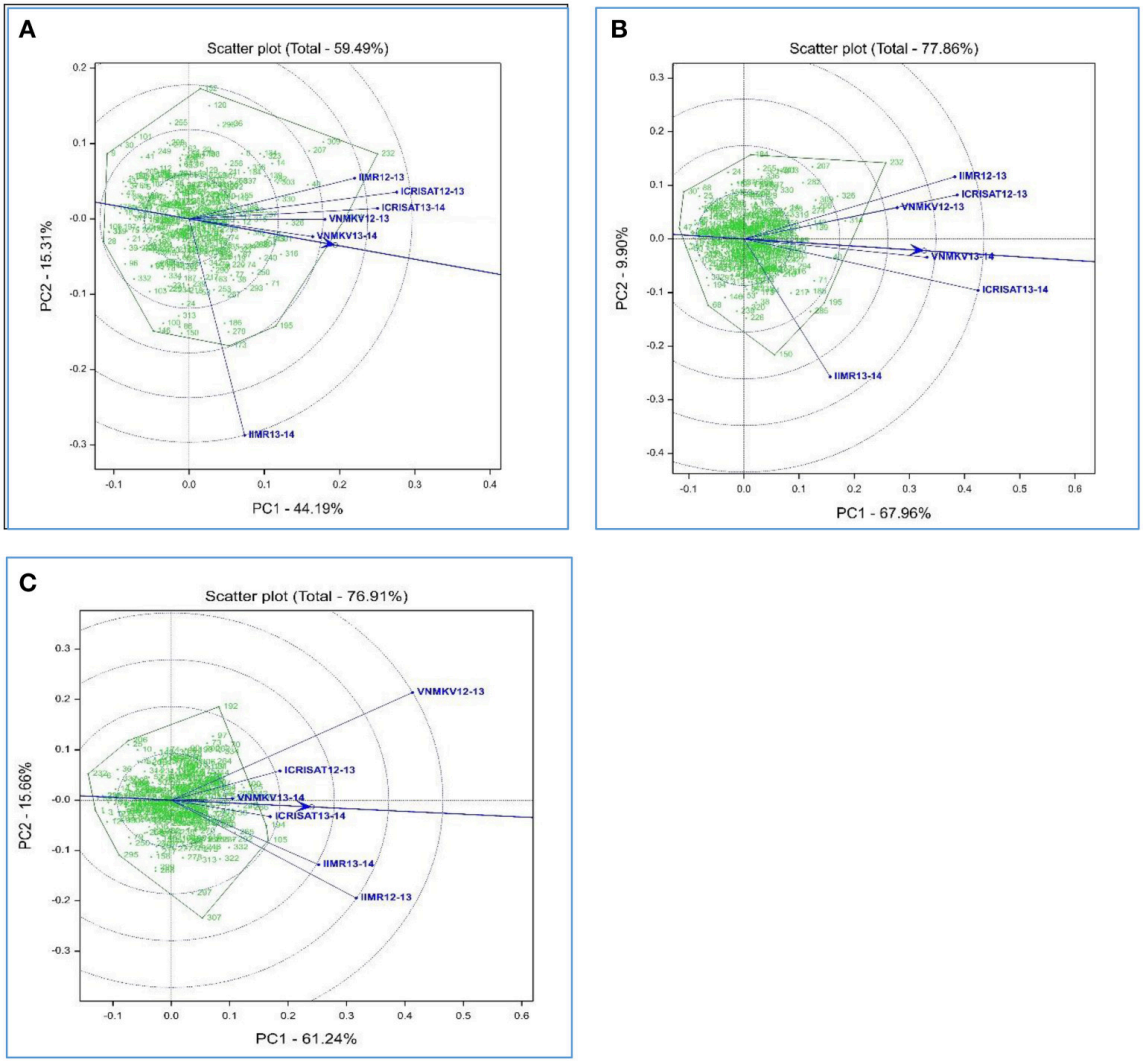
**Heritability-adjusted GGE biplots on six environments for grain iron (A)**, grain zinc **(B)** concentration and grain yield **(C)** performance of 334 recombinant inbred lines in India.

HA- GGE biplots for grain iron concentration revels nearly wide clustering for most of the environments compare to grain zinc concentration, this could be due to low rank correlation between test environment for grain iron concentration i.e., presence of crossover G × E interaction (Table 6A). Environment VNMKV 2013–14 was found to be most representative environment as it has smaller angle with AEA (Average Environment Axis represented by an arrow vector joining center and average co-ordinates of test environment) followed by environment VNMKV 2012–13, ICRISAT 2013–14, ICRISAT 2012–13, and IIMR 2012–13 (Figure 1A). Environment IIMR 2013–14 was most discriminating as having longest vector length, but the angle with AEA was large hence consider as least representative. Also environment IIMR 2013–14 was fall away from remaining all environments which is conform by low rank correlation with other environments. Genotypes G-301, G-326, G-316, and G-143 stands near to AEA in positive direction.

**Table 6:** **Estimation of rank correlation between all six environments**.

**Environment**	**ICRISAT 12-13**	**ICRISAT 13-14**	**IIMR 12-13**	**IIMR 13-14**	**VNMKV 12-13**	**VNMKV 13-14**
**(A) GRAIN IRON CONCENTRATION**						
ICRISAT 12-13	1					
ICRISAT 13-14	0.46^**^	1				
IIMR 12-13	0.39^**^	0.34^**^	1			
IIMR 13-14	0.13^*^	0.11^NS^	0.01^NS^	1		
VNMKV 12-13	0.48^**^	0.40^**^	0.27^**^	0.13^*^	1	
VNMKV 13-14	0.47^**^	0.27^**^	0.30^**^	0.19^**^	0.28^**^	1
**(B) GRAIN ZINC CONCENTRATION**						
ICRISAT 12-13	1					
ICRISAT 13-14	0.64^**^	1				
IIMR 12-13	0.68^**^	0.63^**^	1			
IIMR 13-14	0.34^**^	0.38^**^	0.30^**^	1		
VNMKV 12-13	0.73^**^	0.63^**^	0.68^**^	0.32^**^	1	
VNMKV 13-14	0.70^**^	0.63^**^	0.64^**^	0.41^**^	0.62^**^	1
**(C) GRAIN YIELD**						
ICRISAT 12-13	1					
ICRISAT 13-14	0.40^**^	1				
IIMR 12-13	0.36^**^	0.43^**^	1			
IIMR 13-14	0.45^**^	0.46^**^	0.62^**^	1		
VNMKV 12-13	0.53^**^	0.43^**^	0.50^**^	0.47^**^	1	
VNMKV 13-14	0.52^**^	0.38^**^	0.40^**^	0.52^**^	0.51^**^	1

* Significant at 5% level;

** *Significant at 1% level*.

HA-GGE biplots for grain zinc concentration revels narrow clustering compared to grain iron concentration as there was high rank correlation among test environments (Table 6B). Environment VNMKV 2013–14 was most representative and showed sufficient discrimination for grain zinc concentration followed by ICRISAT 2013–14, VNMKV 2012–13, ICRISAT 2012–13, and IIMR 2012–13 (Figure 1B). Whereas IIMR 2013–14 was least representative as observed in grain iron concentration and stand away from remaining environments due to low rank correlation (Table 6B). Genotype G-40 was most stable as stand near to AEA in positive direction. For both the micronutrient G-232 showed the highest mean value and found stable for environment IIMR 2012–13.

In case of grain yield HA-GGE biplots, it has observed that environment ICRISAT 2013–14 as sufficient discriminating and representative (Figure 1C), hence consider as ideal for selecting stable genotypes followed by ICRISAT 2012–13. Whereas environment VNMKV 2013–14 was least discriminating although showing the smallest angle with AEA. The environment IIMR 2013–14 and IIMR 2012–13 were stand close to each other as having high rank correlation (Table 6C). The genotype G-286 and G-245 were stand close to AEA in positive direction hence consider as most stable genotypes for grain yield.

### Association between grain Fe and Zn concentration and agronomic traits

Pearson's correlation for phenotypic correlation among traits were computed for each individual environment and across environments based on BLUPs of each individual environment and across environments (Table 7). Significant positive and negative correlations were observed between traits studied. Some of the traits were highly correlated while many of them had weak correlations. Based on BLUPs from individual environments, there was highly significant and high positive association between iron and zinc concentration in all environments (E_1_ = 0.79, E_2_ = 0.69, E_3_ = 0.70, E_4_ = 0.72, E_5_ = 0.68, and E_6_ = 0.65; *p* < 0.01) and this trend was consistent in pooled analysis (AE = 0.79; *p* < 0.01). The correlation of grain zinc concentration and grain yield was negative, except in one environment (E_5_) but was smaller in magnitude compared to association between grain iron concentration and grain yield except in one environment (E_6_), whereas iron and zinc concentration were found to be negatively related in same magnitude with grain yield in across the environments analysis. For 100 seed weight, out of six environments grain iron showed significant positive association in three environments (E_1_, E_4_, and E_6_). Whereas, in across environment analysis, iron concentration showed significant positive (AE = 0.34; *p* < 0.01) association with 100 seed weight. In case of zinc concentration, four environments (E_1_, E_2_, E_4_, and E_6_) showed significant positive association and remaining two environments (E_3_ and E_5_) did not show any significant association, whereas for across environment analysis zinc concentration showed significant positive (AE = 0.36; *p* < 0.01) association with 100-seed weight.

Table 7**Estimation of correlation of agronomic trait with grain Fe and Zn contents in sorghum RIL population**.

**Table:** 

	**ICRISAT 12-13 (E1)**			**IIMR 12-13 (E2)**			**VNMKV 12-13 (E3)**		
**Traits**	**Fe**	**Zn**	**GY**	**Fe**	**Zn**	**GY**	**Fe**	**Zn**	**GY**
Zn	0.79^**^	–	–	0.69^**^	–		0.70^**^	–	–
DTF	0.04	−0.06	−0.22^**^	−0.23^**^	−0.10	0.23^**^	0.01	−0.02	−0.01
PH	0.28^**^	0.25^**^	−0.01	0.002	0.12^*^	0.46^**^	0.29^**^	0.30^**^	0.16^**^
TW	0.28^**^	0.30^**^	−0.13^**^	0.06	0.30^**^	0.26^**^	−0.014	−0.023	0.045
GY	−0.31^**^	−0.26^**^	–	−0.27^**^	−0.16^**^	–	−0.21^**^	−0.29^**^	–

**Table:** 

	**ICRISAT 13-14 (E4)**			**IIMR 13-14 (E5)**			**VNMKV 13-14 (E6)**			**Across environment**		
**Traits**	**Fe**	**Zn**	**GY**	**Fe**	**Zn**	**GY**	**Fe**	**Zn**	**GY**	**Fe**	**Zn**	**GY**
Zn	0.72^**^	–	–	0.68^**^	–	–	0.65^**^	–	–	0.79^**^	–	–
DTF	−0.139^*^	−0.11^*^	0.13^**^	−0.077	−0.11^*^	0.12^**^	−0.08^*^	0.08^*^	−0.03	−0.12^*^	−0.12^*^	0.19^**^
PH	0.08	0.22^**^	0.28^**^	0.06	0.03^*^	0.34^**^	0.22^**^	0.31^**^	0.14^**^	0.31^**^	0.33^**^	0.31^**^
TW	0.20^**^	0.40^**^	−0.12^*^	0.06	0.02	−0.01	0.13^*^	0.18^**^	−0.03	0.34^**^	0.36^**^	−0.12^*^
GY	−0.28^**^	−0.23^**^	–	0.06	0.06	–	−0.15^**^	−0.19^**^	–	−0.34^**^	−0.34^**^	–

The association between grain iron concentration and days to 50% flowering ranged from (E_2_ = −0.23; *p* < 0.01 to E1 = 0.04; non-significant), while across the environments shown significant negative (AE = −0.12; *p* < 0.05) association between iron concentration and days to 50% flowering. The association of zinc concentration with days to 50% flowering ranged from (E_4_ = E_5_ = −0.11; *p* < 0.05 to E_6_ = 0.08; *p* < 0.05), while across the environment it showed significant negative (AE = −0.12; *p* < 0.05) association between zinc concentration and days to 50% flowering. The association between plant height and grain iron concentration ranged from (E_2_ = 0.002; non-significant to E_3_ = 0.29; *p* < 0.01), while across environments it showed significant positive (AE = 0.33; *p* < 0.01) association. In case of grain zinc concentration, it ranged from (E_5_ = 0.03; *p* < 0.05 to E_6_ = 0.31; *p* < 0.01), while across environments it showed significant positive association (AE = 0.31; *p* < 0.01). The association between 100 seed weight (seed size) and grain yield ranged from (E_1_ = −0.13, *p* < 0.01 to E2 = 0.26, *p* < 0.01), while across environments it showed significant negative association (AE = −0.12, *p* < 0.05).

## Discussion

The present study was undertaken to understand, first, variability in RIL population for grain Fe and Zn concentration; second, to know the G × E interaction for these two traits; third, association between grain Fe and Zn and other agronomic traits and fourth, to identify stable environment and genotypes for studied traits and based on all results to decide the efficiency of this RIL population for mapping QTLs for grain Fe and Zn concentration.

Present study showed that parents have substantial differences for both micronutrients iron and zinc in all environments. Average iron and zinc was highest in location VNMKV followed by IIMR and ICRISAT respectively as VNMKV soils are deep black and nutrient rich compare to the soils of IIMR and ICRISAT. VNKMV represents heart land of sorghum cultivation in India and both parents used in RIL population development are well adapted to VNMKV environment. In fact male parent PVK 801 was released for commercial cultivation by VNMKV in 2000. This indicated Genotype × Environment interaction and effect of environment in mineral uptake, translocation and distribution. Similar results were obtained by Vreugdenhil et al. (2004) who reported that nutrient content of plant seeds depends on environment factors. Alteration in environment or physiology of plant can affect the accumulation of different multiple elements simultaneously (Buescher, 2010), variation in mineral uptake in different environments has been described in *A. thaliana* (Loudet et al., 2007; Ghandilyan et al., 2009a,b) and *Silene vulgaris* (Ernst et al., 2000). Moreover, the nutrient availability in environment not only affects nutrient concentration of the vegetative, but also of the economic parts of a plant and different soil types also results in micronutrient variability (Ernst et al., 2000). Sankaran et al. (2009) also detected the variation in mineral content in seed between environments and emphasizing importance of environmental factors on quantitative traits. The mean performances results indicated the existence of sufficient variability between parents in overall individual environments and greater opportunities for recovering desirable recombinants by using them in crossing programs. Wide range in within population for micronutrient has also been reported for numerous other crops, for instance in sorghum (Reddy et al., 2005) and maize (Banziger and Long, 2000). Thus availability of wide range for both kind of nutritional and productive traits indicated the scope of development of iron and zinc rich genotypes in high yielding background of sorghum through the exploitation of within population variability, as crop improvement depends on the magnitude of genetic variability in the base population and selection ability. This variability present in population can be exploited if heritability of traits of interest is high. A study conducted in natural populations indicated that the BLUP/REML methodology is a powerful way to estimate the components of variance and to predict additive genetic values (Kruuk, 2004). Interplay between genetic and environmental factors (G × E interactions) affect phenotypes of complex traits which results in reduction in heritability value. G × E interactions also results in different patterns of genetic associations across environments (Ye et al., 2006). Allard and Bradshaw (1964) indicated that the nature of G × E interactions is extremely complex. Detecting G × E interactions requires that the same genotypes are grown in multiple environments in order to allow quantitative genetic analyses. The genetic variance components also play a crucial role in study of heritability. The magnitude of heritability is largely governed by the amount of genetic variance present in the population and high heritability denotes less effect of environment on trait. The analysis of variances for all traits in this experimentation revealed that genotypic variances were highly significant in individual environments as well as across the environments (pooled analysis) indicating high degree of genotypic variance for the traits studied. For agronomic traits the genotype × year × location (σ^2^gyl) interactions values were significant but, lower than genotypic variance, suggesting that the trait are predominantly under genetic control and influenced by environments to a limited extend which implies there is no need for G × E partitioning. Whereas, for both micronutrients, the genotype × year × location (σ^2^gyl) interactions were significant and also higher than genetic variances indicating environment played significant role in grain micronutrient concentration. Assessment of environmental stability of micronutrient is important in crop improvement programs aimed at enhancing the nutritional quality of food crops (Oikeh et al., 2004). The genotype × year × location (σ^2^gyl) interactions for iron were more in magnitude than zinc concentration, similar results were reported earlier by Prasanna et al. (2011) in maize and Gomez-Becerra et al. (2010) in wheat. Suwarto and Nasrullah (2011) found that proportion of G × E interaction was three times higher than genotypic variances for grain iron concentration across eight environments in rice. High G × E interaction for grain iron and zinc concentrations, which affect the rank of genotypes across the environments have been reported in many cereals crops (Banziger and Long, 2000; Oikeh et al., 2003a,b; Oury et al., 2006; Morgonuov et al., 2007; Gomez-Becerra et al., 2010). Studies also showed that Fe and Zn concentration in wheat grain depends largely on environmental conditions, particularly soil availability (Fiel et al., 2005). Therefore iron and zinc concentration in grain show variation according to micronutrients concentration in soil and their availability to plants, more so in wheat another reason for greater G × E interaction for Fe and Zn concentration could be their quantitative inheritance as reported in maize and rice (Gregorio, 2002; Long et al., 2004), through progress in genetic analysis of these traits are expected to be slower than many traits. However, in spite of these challenges there is evidence that breeding for increased levels of micronutrient is feasible (Ortiz-Monasterio et al., 2007; Ashok Kumar et al., 2015).

The HA-GGE biplots are most appropriate of all GGE biplots for graphical evaluation of the test environment as it display the square root heritability (√H^2^) of each test environment based on vector length and its genetic correlation with other test environments (r) based on the angle between two test environments, which are two key elements for test environment evaluation (Yan and Holland, 2010). For grain iron concentration environment VNMKV 2013–14 was most representative hence it is ideal for selecting superior genotypes, whereas IIMR 2013–14 is useful for culling unstable genotypes. In case of grain zinc concentration, environment VNMKV 2013–14 was most representative, hence can be used for selecting ideal genotypes. Whereas IIMR 2013–14 is useful for culling out unstable genotypes. The wide clustering of test environment for grain iron concentration compare to grain zinc concentration indicates high G × E for grain iron than grain zinc concentration. Similarly the contribution of first and second component for grain iron and zinc was 44.19 and 15.31% and, 67.96 and 9.90%, respectively. The locations appeared more unstable for grain iron compared to grain zinc concentration. As the stable location demonstrate a large first primary effect (non-crossover G × E variability) and non-zero secondary effect (crossover G × E variability) in the biplots (Crossa et al., 2002). For both the micronutrients genotype G-232 showed the highest mean value and consistently stable for environment IIMR 2012–13, hence G-232 could be used to develop environment specific cultivar in this particular environment.

For grain yield environment ICRISAT 2013–14 is most representative, hence useful for selecting ideal genotypes, whereas environment VNMKV 2012–13 and IIMR 2012–13 are useful for culling out unstable genotypes. Biplot analysis for grain micronutrients concentration indicated presence of high G × E interaction which proves the instability of environments for micronutrient traits (Fe and Zn), hence while phenotyping these traits special care needs to be taken like (i) use of systematic checks, design like Alpha lattice and spatial analysis of genotypes, (ii) use of micronutrient fertilizers (iron and zinc containing) to homogenize soil iron and zinc concentration.

Development of sorghum cultivars with high levels of grain iron and zinc concentration can make significant contribution to reducing widespread deficiencies of these micronutrients in populations heavily dependent on sorghum for their dietary energy and micronutrient requirements. It is imperative that breeding of such cultivars must not compromise on grain yield and farmer-preferred traits. In present investigation, association between grain iron and zinc showed significant and high positive values and the trend was continued in across environment analysis also. Similar relationships between these micronutrients have been reported in earlier studies on sorghum (Reddy et al., 2005, 2010; Ashok Kumar et al., 2010, 2013; Nguni et al., 2012) and in other cereals, such as pearl millet (Govindaraj et al., 2013; Kanatti et al., 2014), maize (Oikeh et al., 2003a, 2004), rice (Anandan et al., 2011), wheat (Velu et al., 2011), and finger millet (Upadhyaya et al., 2011). These positive associations between iron and zinc densities may likely result from common and overlapping Quantitative Trait Loci (QTL) as reported in wheat (Peleg et al., 2009; Singh et al., 2010), rice (Stangoulis et al., 2007), common bean (Blair et al., 2009; Cichy et al., 2009), and pearl millet (Kumar et al., 2016) implying that simultaneous selection for both micronutrients is likely to be highly effective. This may point to common molecular mechanism controlling the uptake and metabolism of these minerals in grains or common transporters controlling for the minerals (Vreugdenhil et al., 2004; Ghandilyan et al., 2006). Co-segregation of genes for traits might be the reason of strong association between the minerals in both populations. The direction and intensity of association suggested a good possibility of simultaneous genetic improvement of both micronutrients (Velu et al., 2008b) by co-transferring these traits into the elite genetic backgrounds.

The association of grain iron and zinc with grain yield was significantly negative in most of the environment as well as across the environments, but the magnitude of association was low. Such patterns of relationships of grain iron and zinc densities with grain yield are not unexpected considering the high positive correlation between iron and zinc densities and larger G × E interaction effect relative to genotypic effect. Earlier studies in sorghum (Reddy et al., 2005), pearl millet (Rai et al., 2012), wheat (Garvin et al., 2006; Morgonuov et al., 2007; Shi et al., 2008; Zhao et al., 2009), and Maize (Banziger and Long, 2000) also reported significant negative relationship between these micronutrients and grain yield. Through statistically significant (negative), a rather weaker correlation of grain iron content (E_3_ = −0.21 to E_1_ = −0.31) and grain zinc content (E_2_ = −0.16 to E_1_ = −0.26) with yield indicates the possibility of breeding for high iron and zinc concentration in high yielding backgrounds. A good number of sorghum genotypes possessing high yield and high Fe and Zn concentration were developed (Ashok Kumar et al., 2015). This could call for application of genetic tools for selective introgression of only selected genes and genomic regions using marker assisted selection in to the parental lines with high yielding background.

The significant positive association of 100-seed weight with both micronutrients in most of the environment will be more advantageous for selecting simultaneously for the combination of large grain size and high micronutrient traits during genetic improvement program. Whereas non-significant association in sorghum between these two micronutrient and 100 seed weight has been reported earlier (Reddy et al., 2005; Ashok Kumar et al., 2010), while in pearl millet (Velu et al., 2007, 2008a,b) significant positive association was found between grain micronutrient and 100 seed weight. Though the correlation of grain iron and zinc densities with days to 50% flowering and plant height showed significant negative and positive correlation respectively, but the lower magnitude of correlation suggests near-independence of crop growth traits and grain micronutrient traits. The results indicate that sorghum grain iron and zinc concentration can be improved in different maturity and plant stature backgrounds with higher yield.

## Conclusion

Present study on sorghum RIL population showed large variability for both grain micronutrients (iron and zinc), also presence of G × E interaction for both micronutrient indicating influence of environment on the expression of these traits. Compared to zinc, environments showed more influence on grain iron concentration. Further, biplot analysis revealed instability of environment for grain iron and zinc. Therefore, researchers need to take special care while phenotyping of these traits to avoid environmental errors. Constant positive and highly significant correlation between grain iron and zinc concentration showed that simultaneous selection for both the micronutrient will be highly effective. The iron and zinc associated negatively to grain yield but in low magnitude, whereas 100-seed weight showed significant positive association with both the traits in more than 50% of trials and also in across environment analysis indicating cultivars with high iron and zinc can be developed without compensating on grain size. However, while selecting for high iron and zinc densities with high grain yield, large segregating population developed from large number of parental combinations have to be screened than that for yield alone.

The RIL population used in this study showed large variation for all traits studied and even the ranges for all these traits fall outside the parent's values indicating presence of transgressive segregants. In addition, all the traits studied showed high heritabilities indicating superiority of this RIL population for QTL mapping.

## Author contributions

AK: Planned, supervised the research and contributed in preparing the manuscript; RP and KA: Executed the experiments, carried out statistical analysis and prepared manuscript; KR, FJ, GhA, TR, and SD: Supervised the research and manuscript review; KH, SM, and GaA: Helped in phenotyping the RIL population; RD and AR: Helped in statistical analysis; TH and BR: developed RIL population and manuscript review.

### Conflict of interest statement

The authors declare that the research was conducted in the absence of any commercial or financial relationships that could be construed as a potential conflict of interest.

## References

[B1] Allard R. W. Bradshaw A. D. (1964). Implications of genotype-environmental interactions in applied plant breeding. Crop Sci. 4, 503–508. 10.2135/cropsci1964.0011183X000400050021x

[B2] Anandan A. Rajiv G. Eswaran R. Prakash M. (2011). Genotypic variation and relationships between quality traits and trace elements in traditional and improved rice (*Oryzasativa* L.) genotypes. J. Food Sci. 76, 122–130. 10.1111/j.1750-3841.2011.02135.x 22417360

[B3] Ashok Kumar A. Anuradha K. Ramaiah B. Grando S. Frederick H. Rattunde W. (2015). Recent advances in sorghum biofortification research. Plant Breed. Rev. 39, 89–124. 10.1002/9781119107743.ch03

[B4] Ashok Kumar A. Reddy B. V. S. Ramaiah B. Sahrawat K. L. Pfeiffer W. H. (2013). Gene effects and heterosis for grain iron and zinc concentration in sorghum [*Sorghum bicolor* (L.) Moench]. Field Crops Res. 146, 86–95. 10.1016/j.fcr.2013.03.001

[B5] Ashok Kumar A. Reddy B. V. S. Sahrawat K. L. Ramaiah B. (2010). Combating micronutrient malnutrition: Identification of commercial sorghum cultivars with high grain iron and zinc. J. SAT Agric. Res. 8, 1–5.

[B6] Banziger M. Long J. (2000). The potential for increasing the iron and zinc concentration of maize through plant breeding. Food Nutr. Bull. 20, 397–400. 10.1177/156482650002100410

[B7] Black R. E. Allen L. A. Bhutta Z. A. Caulfield L. E. Deonis M. Ezzati M.. (2008). Maternal and child under nutrition: global and regional exposures and health consequences. Lancet 371, 243–260. 10.1016/S0140-6736(07)61690-0 18207566

[B8] Blair M. W. Astudillo C. Grusak M. Graham R. Beebe S. (2009). Inheritance of seed iron and zinc content in common bean (*Phaseolus vulgaris* L.). Mol. Breed. 23, 197–207. 10.1007/s11032-008-9225-z

[B9] Bouis H. E. (2003). Micronutrient fortification of plants through plant breeding: Can it improve nutrition in man at low cost? Proc. Nat. Soc. 62, 403–411. 10.1079/PNS2003262 14506888

[B10] Bouis H. E. Hotz C. McClafferty B. Meenakshi J. V. Pfeiffer W. H. (2011). Biofortification: a new tool to reduce micronutrient malnutrition. Food Nutr. Bull. 32(Suppl. 1), 31–40. 10.1177/15648265110321S105 21717916

[B11] Bouis H. E. Welch R. M. (2010). Biofortification-a sustainable agricultural strategy for reducing micronutrient malnutrition in the global south. Crop Sci. 50, 20–32. 10.2135/cropsci2009.09.0531

[B12] Buescher E. (2010). Natural genetic variation in selected populations of *Arabidopsis thaliana* is associated with ionomic differences. PLoS ONE 5:e11081. 10.1371/journal.pone.0011081 20559418 PMC2885407

[B13] Campbell L. G. Lafever H. N. (1980). Effects of locations and years upon relative yields of the soft red winter wheat region. Crop Sci. 20, 23–28. 10.2135/cropsci1980.0011183X002000010007x

[B14] Cichy K. A. Caldas G. V. Snapp S. S. Blair M. W. (2009). QTL analysis of seed iron, zinc, and phosphorus levels in an Andean bean population. Crop Sci. 49, 1742–1750. 10.2135/cropsci2008.10.0605

[B15] Copenhagen Consensus Centre (2008). Press Release, Copenhagen Consensus Center, Copenhagen Business School, Denmark Available online at: http://www.copenhagenconsensus.com

[B16] Crossa J. Cornelius P. L. Yan W. (2002). Biplot of linear-bilinear models for studying crossover genotype × environment interaction. Crop Sci. 19, 123–132. 10.2135/cropsci2002.1761

[B17] Ernst W. Nelissen H. J M. Ten B. (2000). Combination toxicology of metal-enriched soils: physiological responses of a Zn- and Cd-resistant ecotypes of *Silene vulgaris* on polymetallic soils. Environ. Exp. Bot. 43, 55–71. 10.1016/S0098-8472(99)00048-9

[B18] Ezzati M. Lopez A. D. Rodgers A. Vander H. S. Murray C. J. (2002). Selected major risk factors and global and regional burden of disease. Lancet 360, 1347–1360. 10.1016/S0140-6736(02)11403-6 12423980

[B19] Fiel B. Moser S. Jampatong S. Stamp P. (2005). Mineral composition of the grains of tropical maize varieties as affected by pre-anthesis drought and rate of nitrogen fertilization. Crop Sci. 45, 516–523. 10.2135/cropsci2005.0516

[B20] Fox P. N. Rosielle A. A. (1982). Reducing the influence of environmental main effects on pattern analysis of plant breeding environments. Euphytica 31, 645–656. 10.1007/BF00039203

[B21] Gabriel K. R. (1971). Biplot display of multivariate matrices with application to principal components analysis. Biometrika 58, 453–467. 10.1093/biomet/58.3.453

[B22] Gabriel K. R. (1978). Least squares approximation of matrices by additive and multiplicative models. J. R. Statist. Soc. 40, 186–196.

[B23] Garvin D. F. Welch R. M. Finley J. W. (2006). Historical shifts in the seed mineral micronutrient concentration of US hard red winter wheat germplasm. J. Sci. Food Agric. 86, 2213–2220. 10.1002/jsfa.2601

[B24] Gauch H. G. Zobel R. W. (1996). Predictive and postdictive success of statistical analyses of yield trials. Theor. Appl. Genet. 76, 1–10. 10.1007/BF00288824 24231975

[B25] Ghaderi A. Everson E. H. Cress C. E. (1980). Classification of environments and genotypes in wheat. Crop Sci. 20, 707–710. 10.2135/cropsci1980.0011183X002000060008x

[B26] Ghandilyan A. Barboza L. Tisne S. Grainer C. Reymond M. (2009a). Genetics analysis identifies quantitative trait loci controlling rosette mineral concentrations in *Arabidopsis thaliana* under drought. New Phytol. 184, 180–192. 10.1111/j.1469-8137.2009.02953.x 19656307

[B27] Ghandilyan A. Ilk N. Hanhart C. Mbengue M. Barboza L. (2009b). A strong effect of growth medium and organ type on the identification of QTLs for phytate and mineral concentrations in three *Arabidopsis thaliana* RIL populations. J. Exp. Biol. 60, 1409–1425. 10.1093/jxb/erp084 19346258

[B28] Ghandilyan A. Vreugdenhil D. Aarts M. G. M. (2006). Progress in the genetic under- standing of plant iron and zinc nutrition. Physiol. Plantarum 126, 407–417. 10.1111/j.1399-3054.2006.00646.x

[B29] Gomez-Becerra H. F. Yazici A. Ozturk L. Budak H. Peleg Z. Morgounov A. (2010). Genetic variation and environmental stability of grain mineral nutrient concentrations in Triticum dicoccoides under five environments. Euphytica 171, 39–52. 10.1007/s10681-009-9987-3

[B30] Govindaraj M. Rai K. N. Shanmuga sundaram P. Dwivedi S. L. Sahrawat K. L. Muthaiah A. R. (2013). Combining ability and heterosis for grain iron and zinc densities in pearl millet. Crop Sci. 53, 507–517. 10.2135/cropsci2012.08.0477

[B31] Gregorio G. B. (2002). Progress in breeding for trace minerals in staple crops. J. Nutr. 132, 500–502. 11880579 10.1093/jn/132.3.500S

[B32] Haas J. D. Brownlie T. (2001). Iron deficiency and reduced work capacity: a critical review of the research to determine a causal relationship. J. Nutr. 131, 676–688. 11160598 10.1093/jn/131.2.676S

[B33] Joshi A. K. Mishra B. Chatrath R. Ortiz Ferrara G. Singh R. P. (2007). Wheat improvement in India: present status, emerging challenges and future pro-spects. Euphytica 157, 431–446. 10.1007/s10681-007-9385-7

[B34] Kanatti A. Rai K. N. Radhika K. Govindraj M. Sahrawat K. L. Rao A. S. (2014). Grain iron and zinc concentration in pearl millet: combing ability, hetrosis and association with grain yield and grain size. SpringerPlus 3:763. 10.1186/2193-1801-3-763 25674488 PMC4320223

[B35] Kruuk L. E. B. (2004). Estimating genetic parameters in natural populations using the “animal model.” R. Soc. B. Biol. Sci. 359, 873–890. 10.1098/rstb.2003.1437 15306404 PMC1693385

[B36] Kumar S. Hash C. T. Thirunavukkarasu N. Singh G. Rajaram V. Rathore G.. (2016). Mapping quantative trait loci controlling high iron and zinc content in self and open pollinated grains of pearl millet [*Pennisetum glaucum* (L.) R. Br]. Front. Plant Sci. 7:1636. 10.3389/fpls.2016.01636 27933068 PMC5120122

[B37] Long J. K. Banziger M. Smith M. E. (2004). Diallel analysis of grain iron and zinc density in Southern African-adapted maize inbreds. Crop Sci. 44, 2019–2026. 10.2135/cropsci2004.2019

[B38] Loudet O. Saliba-Colombani V. Camilleri C. Calenge F. Gaudon V. (2007). Natural variation for sulfate content in *Arabidopsis thaliana* is highly controlled by APR2. Nat. Genet. 39, 896–900. 10.1038/ng2050 17589509

[B39] Meenakshi J. V. Johnson N. L. Manyong V. M. Degroote H. Javelosa J. Yanggen D. R. (2010). How cost-effective is biofortification in combating micronutrient malnutrition? An ex- ante assessment. World Develop. 38, 64–75. 10.1016/j.worlddev.2009.03.014

[B40] Morgonuov A. Gomez-Becerra H. F. Abugalieva A. Dzhunusova M. Yessimbekova M. Muminjanov H. (2007). Iron and zinc grain concentration in common wheat grown in Central Asia. Euphytica 155, 193–203. 10.1007/s10681-006-9321-2

[B41] Nguni D. Geleta M. Hofvander P. Fatih M. Bryngelsson T. (2012). Comparative genetic diversity and nutritional quality variation among some important Southern African sorghum accessions [*Sorghum bicolor* (L.) Moench]. Aust. J. Crop Sci. 6, 56–64.

[B42] Oikeh S. O. Menkir A. Maziya-Dixon B. Welch R. Glahn R. P. (2003b). Assessment of concentrations of iron and zinc and bioavailable iron in grains of early-maturing tropical maize varieties. J. Agric. Food Chem. 51, 3688–3694. 10.1021/jf0261708 12769546

[B43] Oikeh S. O. Menkir A. Maziya-Dixon B. Welch R. Glahn R. P. Gauch G. (2004). Environmental stability of iron and zinc concentrations in grain of elite early-maturing tropical maize genotypes grown under field conditions. J. Agric. Sci. 142, 543–551. 10.1017/S0021859604004733

[B44] Oikeh S. O. Menkir A. Maziya-Dixon B. Welch R. M. Glahn R. P. (2003a). Genotypic differences in concentration and bioavailability of kernel-iron intropical maize varieties grown under field conditions. J. Plant Nutr. 26, 2307–2319. 10.1081/PLN-120024283

[B45] Ortiz-Monasterio J. I. Palacios-Rojas N. Meng E. (2007). Enhancing the mineral and vitamin content of wheat and maize through plant breeding. J. Cereal Sci. 46, 293–307. 10.1016/j.jcs.2007.06.005

[B46] Oury F. X. Leenhardt F. Remesy C. Chanliaud E. Duperrier B. Balfourier F. (2006). Genetic variability and stability of grain magnesium, zinc and iron concentrations in bread wheat. Euro. J. Agron. 25, 177–185. 10.1016/j.eja.2006.04.011

[B47] Paterson H. D. Thompson R. (1971). Recovery of inter-block information when block sizes are unequal. Biometrika 58, 545–554. 10.1093/biomet/58.3.545

[B48] Peleg Z. Cakmak I. Ozturk L. Yazici A. Jun Y. Budak H.. (2009). Quantitative trait loci conferring grain mineral nutrient concentrations in durum wheat × wild emmer wheat RIL population. Theor. Appl. Genet. 119, 353–369. 10.1007/s00122-009-1044-z 19407982

[B49] Pfeiffer W. H. McClafferty B. (2007). Harvest plus: breeding crops for better nutrition. Crop Sci. 47, 88–105. 10.2135/cropsci2007.09.0020IPBS

[B50] Prasanna B. M. Mazumdar S. Chakraborti M. Hossain F. Manjaiah K. M. Agrawal P. K. (2011). Genetic variability and genotype × environment interactions for kernel iron and zinc concentrations in maize (*Zea mays*) genotypes. Indian J. Agric. Sci. 81, 704–711.

[B51] Rai K. N. Govindaraj M. Rao A. S. (2012). Genetic enhancement of grain iron and zinc content in pearl millet. Qual. Assur. Safety Crops Foods 4, 119–125. 10.1111/j.1757-837X.2012.00135.x

[B52] Rao P. Birthal B. S. Reddy B. V. S. Rai K. N. Ramesh S. (2006). Diagnostics of sorghum and pearl millet grains-based nutrition in India. Int. Sorghum Millets Newslett. 47, 93–96.

[B53] Reddy B. V. S. Ramesh S. Longvah T. (2005). Prospects of breeding for micronutrients and β-carotene-dense sorghums. Int. Sorghum Millets Newslett. 46, 10–14.

[B54] Reddy P. S. Reddy B. V. S. Ashok Kumar A. Ramesh S. Sahrawat K. L. Rao P. V. (2010). Association of grain Fe and Zn contents with agronomic traits in sorghum. Indian J. Plant Genet. Resour. 23, 280–284.

[B55] Robinson H. F. (1966). Quantitative genetics in relation to breeding on the centennial of mendelism. Indian J. Genet. Plant Breed. 26, 171–187.

[B56] Sankaran R. P. Huguet T. Grusak M. A. (2009). Identification of QTL affecting seed mineral concentrations and content in the model legume Medicago truncatula. Theor. Appl. Genet. 119, 241–253. 10.1007/s00122-009-1033-2 19396421

[B57] Shi R. Li H. Tong Y. Jing R. Zhang F. Zou C. (2008). Identification of quantitative trait locus of zinc and phosphorus density in wheat (*Triticum aestivum* L.) grain. Plant Soil 306, 95–104. 10.1007/s11104-007-9483-2

[B58] Singh K. Chhuneja P. Tiwari V. K. Rawat N. Neelam K. Aggarwal R. (2010). Mapping of QTL for grain iron and zinc content in diploid A genome wheat and validation of these loci in U and S genomes, in Paper Presented at: Plant and Animal Genomes XVIII Conference, (San Diego, CA), 9–13.

[B59] Stangoulis J. C. R. Huynh B. L. Welch R. M. Choi E. Y. Graham R. D. (2007). Quantitative trait loci for phytate in rice grain and their relationship with grain micronutrient content. Euphytica 154, 289–294. 10.1007/s10681-006-9211-7

[B60] Stangoulis J. Sison C. (2008). Crop sampling protocols for micronutrient analysis, in Accounting Principles, 5th Canadian Edn., Vol. 1 (Washington, DC: Harvest Plus Technical Monograph 7).

[B61] Stein A. J. (2010). Global impacts of human mineral malnutrition. Plant Soil 335, 133–154. 10.1007/s11104-009-0228-2

[B62] Stein A. J. Meenakshi J. V. Qaim M. Nestel P. Sachdev H. P. S. Bhutta Z. A. (2008). Potential impacts of iron biofortification in India. Soc. Sci. Med. 66, 1797–1808. 10.1016/j.socscimed.2008.01.006 18291567

[B63] Suwarto Nasrullah (2011). Genotype × environment interaction for iron concentration of rice in central Java of Indonesia. Rice Sci. 18, 75–78. 10.1016/S1672-6308(11)60011-5

[B64] Tesfaye A. (1998). Economics of Milk Production in and Around Hyderabad of Andhra Pradesh. M.Sc. thesis, Acharya NG Ranga Agricultural University, Hyderabad.

[B65] Upadhyaya H. D. Ramesh S. Shivali S. Singh S. K. Varshney S. K. Sarma N. D. R. K. (2011). Genetic diversity for grain nutrients contents in a core collection of finger millet (*Eleusinecoracana* (L.) Gaertn.) germplasm. Field Crops Res. 121, 42–52. 10.1016/j.fcr.2010.11.017

[B66] Velu G. Ortiz-Monasterio I. Singh R. P. Payne T. (2011). Variation for grain micronutrients in wheat core collections accession of diverse origin. Asian J. Crop Sci. 3, 43–48. 10.3923/ajcs.2011.43.48

[B67] Velu G. Rai K. N. Muralidharan V. Kulkarni V. N. Longvah T. Raveendran T. S. (2007). Prospects of breeding biofortified pearl millet with high grain iron and zinc content. Plant Breed. 126, 182–185. 10.1111/j.1439-0523.2007.01322.x

[B68] Velu G. Rai K. N. Sahrawat K. L. (2008b). Variability for grain iron and zinc content in a diverse range of pearl millet populations. Crop Improv. 35, 186–191.

[B69] Velu G. Rai K. N. Sahrawat K. L. Sumalini K. (2008a). Variability for grain iron and zinc contents in pearl millet hybrids. J. SAT Agric. Res. 6, 1–4.

[B70] Vreugdenhil D. Aarts M. G. M. Koornneef M. Nelissen H. Ernst W. H. O. (2004). Natural variation and QTL analysis for cationic mineral content in seeds of *Arabidopsis thaliana*. Plant Cell Environ. 27, 828–839. 10.1111/j.1365-3040.2004.01189.x

[B71] Welch R. M. Graham R. D. (2004). Breeding for micronutrients in staple food crops from a human nutrition perspective. J. Exp. Bot. 55, 353–364. 10.1093/jxb/erh064 14739261

[B72] Wheal M. S. Fowles T. O. Palmer L. T. (2011). A cost-effective acid digestion method using closed polypropylene tubes for inductively coupled plasma optical emission spectrometry (ICP-OES) analysis of plant essential elements. Anal. Methods 3, 2854–2863. 10.1039/c1ay05430a

[B73] World Health Organization (2002). Reducing risks and promoting healthy life. The World Health Report. Geneva: World Health Organization, 168–233.

[B74] White P. J. Broadley M. R. (2005). Biofortifying crops with essential mineral elements. Trends Plant Sci. 10, 586–593. 10.1016/j.tplants.2005.10.001 16271501

[B75] Yan W. Holland J. B. (2010). A heritability-adjusted GGE biplot for test environment evaluation. Euphytica 171, 355–369. 10.1007/s10681-009-0030-5

[B76] Yan W. Sheng Q. Hu Y. G. Hunt L. A. (2001). GGE biplot-an ideal method for graphical analysis of genotype by environment interaction. Acta Agron. Sin. 27, 21–28 (In Chinese).

[B77] Yau S. K. Ortiz-Ferrara G. Srivastava J. P. (1991). Classification of diverse bread wheat-growing environments based on differential yield responses. Crop Sci. 31, 571–576. 10.2135/cropsci1991.0011183X003100030004x

[B78] Ye X. Avendano S. Dekkers J. C. M. Lamont S. J. (2006). Association of twelve immune-related genes with performance of three broiler lines in two different hygiene environments. Poult. Sci. 85, 1555–1569. 16977841 10.1093/ps/85.9.1555

[B79] Zhao F. J. Su Y. H. Dunham S. J. Rakszegi M. Bedo Z. McGrath S. P. (2009). Variation in mineral micronutrient concentrations in grain of wheat lines of diverse origin. J. Cereal Sci. 49, 290–295. 10.1016/j.jcs.2008.11.007

